# Severe Thrombocytopenia and Leukopenia as the Initial Manifestations of Brucella melitensis Bacteremia: A Case Report

**DOI:** 10.7759/cureus.111432

**Published:** 2026-06-24

**Authors:** Carolina Pico Mendoza, Linda E Hernandez Soto, Jose F Gomez Zapata, Luisa F Vazquez Enriquez, Kenia Dominguez Romo

**Affiliations:** 1 Department of Internal Medicine, Hospital General de Zona No. 33, Instituto Mexicano del Seguro Social, Monterrey, MEX; 2 Department of Internal Medicine, Hospital General de Zona No. 67, Instituto Mexicano del Seguro Social, Monterrey, MEX

**Keywords:** bacteremia, brucella melitensis, brucellosis, leukopenia, severe thrombocytopenia

## Abstract

Brucellosis is a zoonotic infection with diverse clinical manifestations that frequently delay diagnosis because of its nonspecific presentation. Hematologic abnormalities have been described in affected patients; however, severe thrombocytopenia remains an uncommon finding. We present the case of a 50-year-old woman who was admitted with a four-day history of fever up to 39°C, diffuse abdominal pain, vomiting, and secretory diarrhea. Initial laboratory evaluation revealed leukopenia (2.7 × 10³/μL), severe thrombocytopenia (29 × 10³/μL), hyponatremia, and elevated liver enzymes. The patient remained hemodynamically stable and showed progressive clinical improvement with supportive management. As part of the etiologic workup, two independent blood cultures yielded *Brucella melitensis*, establishing the diagnosis of systemic brucellosis. Further evaluation identified a history of unpasteurized milk consumption, and the 2-mercaptoethanol test was positive. Treatment with doxycycline and rifampin was initiated, and the patient subsequently demonstrated progressive clinical recovery, recovery of leukocyte and platelet counts, and improvement in biochemical abnormalities. This case highlights the importance of considering brucellosis in the differential diagnosis of patients presenting with acute febrile illness, severe thrombocytopenia, and leukopenia, even in the absence of classic focal manifestations of the disease.

## Introduction

Brucellosis is a zoonotic infection caused by facultative intracellular gram-negative coccobacilli of the genus *Brucella* and remains one of the most common bacterial zoonoses worldwide [1-4]. Human infection is primarily acquired through direct contact with infected animals or the consumption of unpasteurized dairy products. Among the species pathogenic to humans, *Brucella melitensis* is considered the most virulent and is more frequently associated with systemic disease [1-5].

The clinical presentation of brucellosis is highly variable, ranging from a nonspecific febrile illness to focal complications involving the musculoskeletal, neurological, cardiovascular, and hepatobiliary systems. Because of this broad spectrum of manifestations, brucellosis has historically been referred to as "the great imitator" [2,5].

Hematologic abnormalities are frequently observed in brucellosis and may include anemia, leukopenia, thrombocytopenia, and pancytopenia [2,5-8]. Although thrombocytopenia has been reported in approximately 3% to 26% of cases, severe thrombocytopenia remains an uncommon manifestation and has been described primarily in isolated case reports and small case series [7-9]. Because the clinical presentation of brucellosis is often nonspecific, patients with severe thrombocytopenia may initially be misdiagnosed with viral infections, autoimmune disorders, or primary hematologic diseases [6-10]. Furthermore, gastrointestinal symptoms may obscure the diagnosis and contribute to delays in recognition [2,5]. This report highlights an atypical presentation of culture-confirmed *Brucella melitensis* bacteremia, characterized by severe thrombocytopenia and leukopenia in the absence of classic focal manifestations, underscoring the importance of maintaining a high index of suspicion in endemic settings.

We present the case of a patient with *Brucella melitensis* bacteremia whose initial presentation consisted of acute febrile gastrointestinal illness associated with severe thrombocytopenia and leukopenia in the absence of classic focal manifestations of brucellosis.

## Case presentation

A 50-year-old woman with a medical history significant for hepatic steatosis diagnosed in 2023 and a laparoscopic cholecystectomy performed in 2021 presented to the emergency department because of a four-day history of progressively worsening gastrointestinal symptoms. She reported diffuse abdominal pain associated with multiple episodes of non-bloody, food-containing vomiting and frequent secretory diarrhea without dysenteric features. Additionally, she described intermittent fever with documented peaks of up to 39°C, accompanied by malaise and generalized weakness. She denied recent travel, known sick contacts, consumption of undercooked meat, previous similar episodes, mucocutaneous bleeding, epistaxis, gingival bleeding, melena, hematochezia, urinary symptoms, arthralgias, back pain, or focal neurological complaints.

Her past medical history was otherwise unremarkable. She denied diabetes mellitus, hypertension, autoimmune diseases, immunosuppressive conditions, allergies to medications, previous blood transfusions, tobacco use, alcohol consumption, or illicit drug use.

Upon arrival, the patient was hemodynamically stable and did not appear acutely ill. Physical examination revealed an alert and oriented woman in no apparent distress. Cardiopulmonary examination was unremarkable, with normal heart sounds and clear breath sounds bilaterally. The abdomen was soft and depressible, without guarding, rebound tenderness, palpable masses, or evidence of hepatosplenomegaly. No petechiae, ecchymoses, lymphadenopathy, peripheral edema, or other signs suggestive of bleeding or systemic involvement were identified.

Initial laboratory evaluation revealed leukopenia with a white blood cell count of 2.7 × 10³/μL, hemoglobin of 11.3 g/dL, and severe thrombocytopenia with a platelet count of 29 × 10³/μL. Serum chemistry demonstrated hyponatremia (127.9 mmol/L) and hypokalemia (3.26 mmol/L). Liver function tests showed elevated aspartate aminotransferase (AST) of 172 U/L and alanine aminotransferase (ALT) of 136 U/L, while lactate dehydrogenase (LDH) was elevated at 620 U/L. Renal function remained within normal limits (Table 1).

**Table 1:** Evolution of Hematologic and Biochemical Parameters During Hospitalization and Follow-Up Serial laboratory evaluation demonstrated progressive recovery of leukopenia and severe thrombocytopenia during hospitalization and follow-up. Platelet counts increased from 29 × 10³/μL at admission to 223 × 10³/μL during follow-up. Concurrent improvement in leukocyte counts and liver biochemical parameters was observed during follow-up, consistent with progressive clinical recovery. Abbreviations: AST, aspartate aminotransferase; ALT, alanine aminotransferase; LDH, lactate dehydrogenase; "—" indicates that the test was not repeated on the corresponding date.

Parameter	Reference Range	Admission (08/2025)	Hospital Day 2	Hospital Day 3	Hospital Day 4	Discharge	Follow-Up (09/2025)
White blood cell count (×10³/μL)	4.0–10.0	2.7	3.2	4.8	4.7	4.4	5.4
Hemoglobin (g/dL)	12.0–16.0	11.3	10.1	11.5	11.5	10.6	10.8
Platelet count (×10³/μL)	150–450	29	30	47	53	61	223
Sodium (mmol/L)	136–145	127.9	138.6	136.4	—	—	—
Potassium (mmol/L)	3.5–5.1	3.26	3.57	3.83	—	—	—
Creatinine (mg/dL)	0.50–0.90	0.78	0.46	0.44	—	0.43	0.54
AST (U/L)	10–35	172	—	—	—	98	39.4
ALT (U/L)	10–35	136	—	—	—	96	35.9
LDH (U/L)	125–220	620	—	—	—	357	—

Given the severity of thrombocytopenia, the patient was admitted for close clinical monitoring and serial laboratory assessment. Despite the markedly reduced platelet count, she remained free of hemorrhagic manifestations throughout hospitalization. Progressive improvement in leukocyte and platelet counts was documented on subsequent complete blood count analyses.

As part of the diagnostic evaluation, abdominopelvic computed tomography without intravenous contrast was performed. Imaging demonstrated diffuse hepatic steatosis without evidence of hepatosplenomegaly, intra-abdominal lymphadenopathy, abscess formation, bowel wall abnormalities, free fluid, or other focal lesions that could explain the patient's clinical presentation.

Because of the persistent febrile syndrome associated with hematologic abnormalities, blood cultures and additional microbiological studies were obtained during hospitalization. Two independent blood culture sets subsequently yielded growth of *Brucella melitensis*, thus establishing the diagnosis of systemic brucellosis. Following microbiological confirmation and Infectious Diseases consultation, a history of unpasteurized milk consumption was identified, representing the most likely source of infection. Furthermore, the 2-mercaptoethanol test was positive, providing additional evidence supportive of active disease.

After confirmation of the diagnosis, targeted antimicrobial therapy with doxycycline 100 mg administered orally every 12 hours in combination with rifampin was initiated, with a planned treatment duration of six weeks. The patient exhibited progressive clinical improvement, characterized by resolution of fever and gastrointestinal symptoms, recovery of leukocyte and platelet counts, and gradual improvement of liver biochemical abnormalities. Follow-up laboratory testing demonstrated complete resolution of thrombocytopenia, with platelet counts increasing to 223 × 10³/μL, consistent with a favorable clinical course.

## Discussion

Brucellosis remains a significant public health concern in many regions of the world because of its broad geographic distribution and diverse clinical manifestations [1-5]. Human infection is most commonly acquired through direct contact with infected animals or the consumption of unpasteurized dairy products. In our patient, a history of unpasteurized milk consumption was identified following consultation with the Infectious Diseases service, representing the most likely source of infection.

The clinical presentation of brucellosis is highly variable and frequently nonspecific, ranging from acute febrile illness to focal complications involving the musculoskeletal, neurological, cardiovascular, and hepatobiliary systems [2,5]. Because of this heterogeneity, brucellosis has traditionally been referred to as "the great imitator." Our patient initially presented with fever, diffuse abdominal pain, vomiting, and secretory diarrhea, findings that are more suggestive of acute gastrointestinal infection than systemic brucellosis. Furthermore, the absence of osteoarticular manifestations, hepatosplenomegaly, and lymphadenopathy contributed to the diagnostic challenge.

Hematologic abnormalities have been increasingly recognized in patients with brucellosis and may include anemia, leukopenia, thrombocytopenia, and pancytopenia [5-7]. Although thrombocytopenia is not uncommon, severe thrombocytopenia remains a relatively rare manifestation of brucellosis and has been reported primarily in isolated case reports and small case series [7-9]. Several mechanisms have been proposed to explain these abnormalities, including hypersplenism, immune-mediated peripheral destruction, bone marrow granulomatous involvement, hemophagocytosis, and disseminated intravascular coagulation [6,7]. In the present case, the platelet count at admission was 29 × 10³/μL, representing one of the most clinically relevant findings. Despite the severity of thrombocytopenia, no hemorrhagic manifestations were observed throughout hospitalization.

Previous reports have described severe thrombocytopenia as the initial manifestation of brucellosis, occasionally mimicking immune thrombocytopenic purpura or other primary hematologic disorders [7-9]. Similar to those cases, our patient presented with severe thrombocytopenia accompanied by leukopenia and mild anemia, a constellation of findings that may initially prompt consideration of viral infections, autoimmune diseases, or hematologic malignancies rather than zoonotic bacterial infection.

The diagnosis of brucellosis may be established using serologic assays, molecular methods, or microbiological isolation. However, recovery of the organism from blood cultures remains an important confirmatory tool for active infection [3]. In this case, two independent blood culture sets yielded growth of *Brucella melitensis*, providing definitive microbiological confirmation and strengthening the diagnostic value of this report. The positive 2-mercaptoethanol test provided additional evidence supporting active disease.

Peripheral blood smear review and bone marrow examination were not performed during hospitalization. Although these investigations may be useful in excluding alternative causes of severe thrombocytopenia, the diagnosis of active brucellosis was microbiologically confirmed by two independent blood cultures yielding *Brucella melitensis*. Furthermore, the patient demonstrated progressive clinical and hematologic recovery during follow-up, supporting the overall diagnostic assessment and favorable clinical course.

Current therapeutic recommendations support the use of combination antimicrobial therapy to reduce relapse rates and achieve microbiological eradication [10]. Our patient received doxycycline and rifampin for a planned six-week course and subsequently demonstrated progressive clinical improvement, recovery of leukocyte and platelet counts, and improvement of biochemical abnormalities. As demonstrated in Table 1, platelet recovery paralleled the patient's overall clinical improvement.

This case highlights the importance of maintaining a high index of suspicion for brucellosis in patients presenting with acute febrile illness associated with severe thrombocytopenia and leukopenia, even in the absence of classic focal manifestations. Early recognition of atypical presentations may prevent diagnostic delays and facilitate timely initiation of appropriate antimicrobial therapy.

## Conclusions

Brucellosis should be considered in the differential diagnosis of acute febrile illness accompanied by severe thrombocytopenia and leukopenia, even in the absence of classic focal manifestations.

This case highlights that *Brucella melitensis* bacteremia may initially present with predominantly gastrointestinal symptoms and significant hematologic abnormalities. Early recognition of these atypical presentations and prompt initiation of appropriate antimicrobial therapy may contribute to favorable clinical outcomes and hematologic recovery.
